# Intracranial lesion features in moderate-to-severe traumatic brain injury: relation to neurointensive care variables and clinical outcome

**DOI:** 10.1007/s00701-023-05743-y

**Published:** 2023-08-08

**Authors:** Teodor Svedung Wettervik, Anders Hånell, Per Enblad, Anders Lewén

**Affiliations:** Department of Medical Sciences, Section of Neurosurgery, Uppsala University, 751 85 Uppsala, Sweden

**Keywords:** Cerebral perfusion pressure, Hemorrhage progression, Intracranial pressure, Neurointensive care, Pressure reactivity index, Traumatic brain injury

## Abstract

**Background:**

The primary aim was to determine the association of intracranial hemorrhage lesion type, size, mass effect, and evolution with the clinical course during neurointensive care and long-term outcome after traumatic brain injury (TBI).

**Methods:**

In this observational, retrospective study, 385 TBI patients treated at the neurointensive care unit at Uppsala University Hospital, Sweden, were included. The lesion type, size, mass effect, and evolution (progression on the follow-up CT) were assessed and analyzed in relation to the percentage of secondary insults with intracranial pressure > 20 mmHg, cerebral perfusion pressure < 60 mmHg, and cerebral pressure autoregulatory status (PRx) and in relation to Glasgow Outcome Scale-Extended.

**Results:**

A larger epidural hematoma (*p* < 0.05) and acute subdural hematoma (*p* < 0.001) volume, greater midline shift (*p* < 0.001), and compressed basal cisterns (*p* < 0.001) correlated with craniotomy surgery. In multiple regressions, presence of traumatic subarachnoid hemorrhage (*p* < 0.001) and intracranial hemorrhage progression on the follow-up CT (*p* < 0.01) were associated with more intracranial pressure-insults above 20 mmHg. In similar regressions, obliterated basal cisterns (*p* < 0.001) were independently associated with higher PRx. In a multiple regression, greater acute subdural hematoma (*p* < 0.05) and contusion (*p* < 0.05) volume, presence of traumatic subarachnoid hemorrhage (*p* < 0.01), and obliterated basal cisterns (*p* < 0.01) were independently associated with a lower rate of favorable outcome.

**Conclusions:**

The intracranial lesion type, size, mass effect, and evolution were associated with the clinical course, cerebral pathophysiology, and outcome following TBI. Future efforts should integrate such granular data into more sophisticated machine learning models to aid the clinician to better anticipate emerging secondary insults and to predict clinical outcome.

## Introduction

Traumatic brain injury (TBI) leads to a significant burden of mortality and morbidity worldwide [4, 30]. The post-traumatic intracranial hemorrhagic lesion type, size, mass effect, and evolution significantly affect the clinical course and outcome for the individual patient [13, 17, 18, 29]. Neurosurgical care in TBI is devoted to detection and emergency evacuation of significant intracranial hemorrhages to avoid brain herniation and neurointensive care (NIC) to improve the cerebral environment by managing systemic and cerebral physiology [11, 22]. Specifically, elevated intracranial pressure (ICP), low cerebral perfusion pressure (CPP), and disturbances in cerebral pressure autoregulation (PRx) are important predictors of poor outcome and are also potentially treatable insults during NIC [6, 10, 27]. There is a general understanding that large traumatic intracranial hemorrhages contribute to a more complicated clinical course and contribute to worse derangements in cerebral physiology (ICP, CPP, and PRx), both preoperatively due to the volume effect of the lesion as well as postoperatively, e.g., related to secondary brain swelling. However, only a few studies [34, 35] have in detail investigated the association of specific intracranial hemorrhagic types (epidural hematoma (EDH), acute subdural hematoma (ASDH), subarachnoid hemorrhage (SAH), intraventricular hemorrhage (IVH), and contusions) and their sizes, on the monitoring and treatment variables of the acute phase in the NIC. This is of interest in order to better understand how in-depth analyses of early radiological imaging data may be used to understand the immediate clinical course following moderate to severe TBI, which is often challenging to predict. In addition, attentive NIC strives to keep physiological variables within optimal ranges to avoid secondary brain insults [11, 22] and it remains to be determined to what extent early intracranial hemorrhage features affect the success rate of keeping the patients within these NIC targets. Thus, by means of granular volumetric intracranial hemorrhage assessments, detailed information of the clinical course, and high-frequency physiological data from the NIC in a large cohort of TBI patients, we aimed to explore the role of specific intracranial hemorrhage features (type of lesion, volumes, mass effect, and evolution) on the clinical course, derangements in cerebral physiology (ICP, CPP, and PRx) during NIC, and long-term clinical outcome. We hypothesized that large lesions of particularly EDHs, ASDHs, and contusions would require emergency evacuation, but would not lead to more ICP and CPP insults since these variables were actively optimized during vigilant NIC. PRx was not actively treated and was hypothesized to be more disturbed in association to larger hemorrhage volumes and specifically related to SAH and ASDHs [1, 34].

## Materials and methods

### Patients and study design

This was a retrospective analysis of prospectively collected data, conducted at the Department of Neurosurgery at the Uppsala University Hospital, Sweden. There were 514 TBI patients aged 16 or older who received ICP-monitoring at our NIC unit between 2008 and 2018. Four hundred seventy-two of these had long-term outcome data, 409 patients had at least 12 h of available ICP data the first 7 days post-injury, and those 385 that also had two available computed tomography (CT) scans (and the 2nd within 48 h from the first scan) made up the final study cohort.

### Neurointensive care management protocol

The patients were treated according to our standardized ICP- and CPP-oriented TBI management protocol, which has been described in detail in previous studies [11]. Treatment goals included ICP ≤ 20 mmHg, CPP ≥ 60 mmHg, systolic blood pressure ≥ 100 mmHg, pO_2_ ≥ 12 kPa, arterial glucose 5–10 mM, hemoglobin ≥ 10 g/dL, electrolytes within normal ranges, normovolemia, and body temperature < 38 °C.

Unconscious (GCS M 1–5) patients were intubated, mechanically ventilated, and received propofol and morphine for sedation and analgesia, respectively. ICP was monitored with an external ventricular catheter (EVD; HanniSet, Xtrans, Smith Medical GmbH, Glasbrunn, Germany) or an intraparenchymal sensor device (Codman ICP Micro-Sensor, Codman & Shurtleff, Raynham, MA) in all unconscious patients. Surgical evacuation was done in case of significant intracranial mass lesions. Basal ICP-lowering treatments included head elevation to 30° and mild hyperventilation (pCO_2_ 4.0–4.5 kPa). Neurological wake-up tests were done three times per day. In situations of increased ICP, despite basic treatment and if no mass lesion was present, an EVD was used to drain cerebrospinal fluid. If ICP was still refractory elevated, the patients were held continuously sedated and stress was treated with increased sedation, pain relief, β_1_-antagonists, and/or α_2_-agonists. If the patients did not respond to these treatments, last-tier treatments included thiopental infusion (if there was no midline shift and uncus herniation) and/or decompressive craniectomy (DC) [31].

### Data acquisition and analysis

The physiological variables (arterial blood pressure (ABP) and ICP) were recorded at 100 Hz using the Odin software [15]. ABP was measured in the radial artery at heart level. ICP was monitored with either an EVD or intraparenchymal probe, as outlined above. PRx was calculated as the 5 min correlation of 10 s averages of ICP and mean arterial blood pressure [10, 26]. PRx was calculated in retrospect and was not available bedside. The percentage of monitoring time with ICP (> 20 mmHg) and CPP insults (< 60 mmHg) as well as mean PRx were calculated in Odin for the first 7 days post-injury. The insult thresholds were defined in accordance with our management protocol, whereas we assessed mean PRx since specific thresholds for this variable are less established and we also had no specific treatment target for this variable.

The initial post-traumatic CT scan of the brain was evaluated according to the Marshall classification [18] for each patient. Volumetric (mL) assessments of the intracranial hemorrhagic lesion types (EDH, ASDH, IVH, and contusions) were evaluated and compared on the first two CT scans in the Brainlab software. tSAH volumes could not be calculated reliably and these lesions were only defined as present or absent. Hemorrhage progression of intracranial lesions was defined as an increase in EDH/ASDH/IVH/contusions with 6 mL or more, in accordance with previous studies [23, 25]. Some patients received emergency neurosurgery immediately after the first CT and to take into account these different clinical trajectories of hemorrhage/injury evolution, the patients were divided into three groups: (1) stable intracranial hemorrhages on follow-up CT, (2) progression of intracranial hemorrhages on follow-up CT as defined above, (3) immediate intracranial surgery after the first CT. Midline shift (mm) and basal cisterns (open/compressed/obliterated) were assessed on both the 1st and 2nd CT. The largest volume for each lesion and the most severe mass effect on any of the 1st or 2nd CT scan was used for each patient in the statistical analyses below.

### Outcome

Clinical outcome was assessed by specially trained personnel with structured telephone interviews at 6 months post-injury using the Extended Glasgow Outcome Scale (GOS-E), which has eight categories of global outcome, from death to upper good recovery [28, 32]. The interviews were held with the patients if they had recovered sufficiently, otherwise with their next of kin.

### Statistical analysis

The primary aim was (i) to determine the association between intracranial hemorrhage lesion type, size, mass effect, and evolution in relation to the clinical course (ICP-lowering treatments and NIC variables) and secondarily (ii) in relation to clinical outcome.

Demography, admission status, treatments, radiological measures, NIC variables, and clinical outcome were described as median (interquartile range) or number (proportion), depending on the type of data. The association between EDH (mL), ASDH (mL), tSAH (yes/no), IVH (mL), contusions (mL), midline shift (mm), and basal cisterns (assessed as an ordinal measure; open/compressed/obliterated) with ICP-lowering treatments (craniotomy, thiopental, and DC), NIC variables (mean PRx and monitoring time: ICP > 20 mmHg, CPP < 60 mmHg), and clinical outcome (GOS-E) was analyzed with the Spearman correlation coefficient. The hematoma volume was denoted as 0 mL in the analyses if the patient did not have that type of intracranial hemorrhagic lesion type. The association between hemorrhage evolution (stable, progression, emergency neurosurgery) was analyzed in relation to the same variables by using the chi-square or Kruskal-Wallis test, depending on the data (categorical/ordinal/continuous). Separate multiple linear regression with ICP > 20 mmHg, CPP < 60 mmHg, and PRx, respectively, as dependent variables and age, GCS M, pupillary status, EDH (mL), ASDH (mL), tSAH (yes), IVH (mL), contusion (mL), midline shift (mm), basal cisterns (open/compressed/obliterated), and intracranial hemorrhage evolution (progression and emergency neurosurgery) as independent variables were done. In these separate regressions, the percentage of ICP > 20 mmHg and CPP < 60 mmHg were log_10_-transformed due to skewness of data. Similarly, a multiple logistic regression analysis for favorable outcome was done with age, GCS M, pupillary status, EDH (mL), ASDH (mL), tSAH (yes), IVH (mL), contusion (mL), midline shift (mm), basal cisterns (open/compressed/obliterated), and intracranial hemorrhage evolution (progression and emergency neurosurgery) as independent variables. Missing data were rare and these few cases were excluded from the analyses. A *p*-value < 0.05 was considered statistically significant. The statistical analyses were performed in SPSS version 28 (IBM Corp, Armonk, NY, USA).

## Results

### Demography, admission variables, treatments, and clinical outcome

In this cohort of 385 TBI patients (Table 1), median age was 52 (IQR 31–65) years and the male/female ratio was 300/85 (78/22%). GCS M was in median 5 (IQR 4–5) and 76 (19%) cases exhibited a pupillary abnormality (unreactive) at NIC admission. Marshall score was in median 2 (IQR 2–5), 201 (52%) underwent a craniotomy, 49 (13%) received thiopental infusion, and 43 (11%) were operated with a DC. Eleven patients died within the first 7 days due to intracranial hypertension. At 6 months post-injury, median GOS-E was 5 (IQR 3–7), 203 (53%) had recovered favorably, and 63 (16%) were deceased.

**Table 1:** Demographics, admission variables, treatments, and clinical outcome

Patients, *n* (%)	385 (100%)
*Demography*	
Age (years), median (IQR)	52 (31–65)
Sex (male/female), *n* (%)	300/85 (78/22%)
Antithrombotic agents (no AT/AP/AC/DAT), *n* (%)	321/28/25/11 (83/7/6/3%)
*Admission variables*	
GCS M score at admission (1–4/5–6), *n* (%)	108/277 (28/72%)
Pupillary abnormalities (yes), *n* (%)	76 (20%)
Marshall CT classification (diffuse injury I–IV/focal lesion grade V–VI, *n* (%)	253/132 (66/34%)
*Monitoring and treatments*	
ICP monitor (Codman/EVD/both), *n* (%)	218/77/90 (57/20/23%)
Craniotomy/hematoma evacuation (yes), *n* (%)	201 (52%)
Thiopental (yes), *n* (%)	49 (13%)
DC (yes), *n* (%)	43 (11%)
*Outcome*	
Favorable/unfavorable outcome (GOS-E 5–8/1–4), *n* (%)	203/182 (53/47%)
Mortality, *n* (%)	63 (16%)

*AC* anticoagulant, *AP* antiplatelet, *AT* antithrombotic agents, *CT* computed tomography, *DAT* dual antithrombotic agents, *DC* decompressive craniectomy, *EVD* external ventricular drainage, *GCS M* Glasgow Coma Scale Motor, *GOS-E* Glasgow Outcome Scale-Extended, *ICP* intracranial pressure, *IQR* interquartile range

### Intracranial hemorrhage type, size, mass effect, and evolution

On any of the first two CT scans, 34 (9%) exhibited an EDH, 170 (44%) an ASDH, 279 (72%) tSAH, 94 (24%) IVH, and 272 (71%) contusions. The maximal size of the hemorrhage as well as the extent of midline shift and compression of the basal cisterns on any of the two first CT scans is described in Table 2. Almost half (*n* = 186 (48%)) of the cohort exhibited a stable hemorrhage on the follow-up CT, whereas 108 (28%) showed significant hemorrhage progression, and 91 (24%) required emergency neurosurgery after the first CT.

**Table 2:** Intracranial hemorrhagic lesion type, volume, mass effect, and evolution

*Intracranial hemorrhagic lesion type and volume*	
EDH (yes), *n* (%)	34 (9%)
EDH size (mL), median (IQR)	23 (14–60)
ASDH (yes), *n* (%)	170 (44%)
ASDH size (mL) at first CT, median (IQR)	22 (8–81)
tSAH (yes), *n* (%)	279 (72%)
IVH (yes), *n* (%)	94 (24%)
IVH size (mL), at first CT, median (IQR)	0.6 (0.2–1.9)
Contusion (yes), *n* (%)	272 (71%)
Contusion size (mL), at first CT, median (IQR)	7 (2–26)
*Mass effect*	
Midline shift (mm), median (IQR)	0 (0–7)
*Evolution*	
Basal cisterns (open/compressed/obliterated), *n* (%)	315/50/20 (82/13/5%)
Intracranial hemorrhage evolution (stable, hemorrhagic progression, immediate surgery), *n* (%)	186/108/91 (48/28/24%)

In the calculations of median and IQR for the volumes of the various intracranial hemorrhagic lesions types, only those with such a lesion were included

*ASDH* acute subdural hematoma, *EDH* epidural hematoma, *IQR* interquartile range, *IVH* intraventricular hemorrhage, *tSAH* traumatic subarachnoid hemorrhage

### Cerebral physiology the first 7 days after TBI

During the first 7 days post-injury, ICP was in median 12 (IQR 9–15) mmHg and the percentage of monitoring time with ICP above 20 mmHg was in median 3 (IQR 1–10) %. During the same period, median CPP was 75 (IQR 71–82) mmHg, whereas the percentage of monitoring time of CPP below 60 mmHg was in median 4 (IQR 1–9) %. PRx was in median 0.06 (IQR − 0.03–0.14).

### Intracranial hemorrhage type, size, mass effect, and evolution in relation to ICP-lowering treatments and cerebral physiology

In univariate analyses (Table 3), patients with larger EDH (*r* = 0.10, *p* < 0.05), ASDH (*r* = 0.51, *p* < 0.001), greater midline shift (*r* = 0.64, *p* < 0.001), and more compressed basal cisterns (*r* = 0.39, *p* < 0.001) were more often operated with craniotomy, whereas presence of tSAH (*r* =  − 0.10, *p* < 0.05) and larger IVH size (*r* =  − 0.18, *p* < 0.001) showed the opposite association. Greater contusion size (*r* = 0.10, *p* < 0.05) correlated with the need for thiopental (*r* = 0.10, *p* < 0.05), whereas tSAH (*r* = 0.13, *p* < 0.05), contusion size (*r* = 0.10, *p* < 0.05), and more compressed basal cisterns (*r* = 0.21, *p* < 0.001) were associated with DC surgery. The same variables (tSAH, contusion volume, and more compressed basal cisterns) were also associated with more ICP insults. Greater EDH volume (*r* = 0.16, *p* < 0.01) and no/smaller ASDH (*r* =  − 0.15, *p* < 0.01) were associated with more CPP insults below 60 mmHg. Larger ASDH volume (*r* = 0.24, *p* < 0.001), midline shift (*r* = 0.24, *p* < 0.001), and compressed basal cisterns (*r* = 0.18, *p* < 0.001) were associated with higher PRx.

**Table 3:** Association between intracranial hemorrhage lesion type and size with clinical course, neurointensive care variables, and clinical outcome—a Spearman correlation analysis

	Craniotomy (yes)	Thiopental (yes)	DC (yes)	ICP > 20 mmHg (%)	CPP < 60 mmHg (%)	PRx (coefficient)	GOS-E (scale)
EDH (mL)	***0.10***^***a***^	− 0.06	− 0.08	0.10	***0.16***^***b***^	− 0.05	***0.20***^***c***^
ASDH (mL)	***0.51***^***c***^	− 0.06	0.05	− 0.04	− ***0.15***^***b***^	***0.24***^***c***^	− ***0.19***^***c***^
tSAH (yes)	− ***0.10***^***a***^	0.10	***0.13***^***a***^	***0.20***^***c***^	0.05	− 0.03	− ***0.13***^***a***^
IVH (mL)	− ***0.18***^***c***^	− 0.01	− 0.05	− 0.07	− 0.08	0.05	− ***0.13***^***a***^
Contusion (mL)	0.08	***0.10***^***a***^	***0.10***^***a***^	***0.24***^***c***^	0.08	0.05	− ***0.17***^***c***^
Midline shift (mm)	***0.64***^***c***^	− 0.05	0.09	0.00	− 0.09	***0.24***^***c***^	− ***0.18***^***c***^
Basal cisterns*	***0.39***^***c***^	0.07	***0.21***^***c***^	***0.11***^***a***^	0.08	***0.18***^***c***^	− ***0.23***^***c***^

Bold and italics indicate statistical significance

The hematoma volume was denoted as 0 mL in case the analyses if the patient did not have that type of intracranial hemorrhagic lesion type

*ASDH* acute subdural hematoma, *CPP* cerebral perfusion pressure, *DC* decompressive craniectomy, *EDH* epidural hematoma, *GOS-E* Glasgow Outcome Scale-Extended, *ICP* intracranial pressure, *IVH* intraventricular hemorrhage, *PRx* pressure reactivity index, *tSAH* traumatic subarachnoid hemorrhage

^a^*p* < 0.05; ^b^*p* < 0.01; ^c^*p* < 0.001

^*^The extent of basal cistern compression was assessed as an ordinal variable (1/open, 2/compressed, 3/obliterated)

Those with a stable follow-up CT were operated with a craniotomy and DC to a lesser extent than those with hemorrhage progression or emergency neurosurgery after the first CT (*p* < 0.01; Table 4). Those with a stable CT also exhibited a lower percentage of ICP insults and mean PRx (*p* < 0.01).

**Table 4:** Association between intracranial hemorrhage evolution with clinical course, neurointensive care variables, and clinical outcome

Variables		Intracranial hemorrhage evolution			
		Stable	Hemorrhage progression	Emergency neurosurgery	*p*-value
Craniotomy	**Yes, *n* (%)**	***48 (26%)***	***62 (57%)***	***91 (100%)***	***0.001***
	**No, *n* (%)**	***138 (74%)***	***46 (43%)***	***0 (0%)***	
Thiopental	**Yes, *n* (%)**	24 (13%)	17 (16%)	8 (9%)	0.34
	**No, *n* (%)**	162 (87%)	91 (84%)	83 (91%)	
DC	**Yes, *n* (%)**	***13 (7%)***	***13 (12%)***	***17 (19%)***	***0.01***
	**No, *n* (%)**	***173 (93%)***	***95 (88%)***	***74 (81%)***	
ICP > 20 mmHg (%), median (IQR)		***3 (0–10)***	***5 (2–14)***	***3 (1–11)***	***0.01***
CPP < 60 mmHg (%), median (IQR)		4 (1–9)	4 (1–8)	3 (1–11)	0.91
PRx, median (IQR)		***0.02 (− 0.07–0.10)***	***0.07 (0.00–0.17)***	***0.09 (0.01–0.19)***	***0.001***
GOS-E, median (IQR)		***5 (3–7)***	***5 (3–7)***	***3 (2–7)***	***0.009***

*ASDH* acute subdural hematoma, *CPP* cerebral perfusion pressure, *DC* decompressive craniectomy, *EDH* epidural hematoma, *GOS-E* Glasgow Outcome Scale-Extended, *ICP* intracranial pressure, *IQR* interquartile range, *IVH* intraventricular hemorrhage, *PRx* pressure reactivity index, *tSAH* traumatic subarachnoid hemorrhage

In multiple linear regression analyses (Table 5), presence of tSAH (β = 0.20, *p* < 0.001), no/smaller volumes of IVH (β =  − 0.14, *p* < 0.001), and hemorrhage progression on follow-up CT (β = 0.17, *p* < 0.01) together with younger age (β =  − 0.29, *p* < 0.001) were independently associated with a greater burden of ICP insults above 20 mmHg. Younger age (β =  − 0.35, *p* < 0.001), no/smaller volumes of ASDH (β =  − 0.29, *p* < 0.001), hemorrhage progression on the second CT (β = 0.14, *p* < 0.05), and emergency neurosurgery (β = 0.25, *p* < 0.001) after the first CT were independently associated with a higher burden of CPP < 60 mmHg. Lastly, higher age (β = 0.14, *p* < 0.05), lower GCS M at admission (β =  − 0.20, *p* < 0.001), and more compressed/obliterated basal cisterns (β = 0.18, *p* < 0.01) were associated with higher PRx.

**Table 5:** Intracranial hemorrhage lesion types, size, mass effect, and evolution in relation to ICP, CPP, and PRx—multiple linear regression analyses

Variables	ICP > 20 mmHg (%)	CPP < 60 mmHg (%)	PRx (coefficient)
Age (years)	*** − 0.29***^***c***^	*** − 0.35***^***c***^	***0.14***^***a***^
GCS M (scale)	*** − ***0.08	*** − ***0.03	*** − 0.20***^***c***^
Pupillary abnormality (yes)	0.03	0.01	0.04
EDH (mL)	0.03	*** − ***0.10	*** − ***0.02
ASDH (mL)	0.03	*** − 0.29***^***c***^	0.01
tSAH (yes)	***0.20***^***c***^	0.05	0.03
IVH (mL)	*** − 0.14***^***b***^	*** − ***0.09	0.03
Contusion (mL)	0.11	*** − ***0.02	0.05
Midline shift (mm)	0.02	0.07	0.08
Basal cisterns*	0.04	0.09	***0.18***^***b***^
Intracranial hemorrhage evolution			
Progression (yes)	***0.17***^***b***^	***0.14***^***a***^	0.04
Evacuation (yes)	0.12	***0.25***^***c***^	*** − ***0.07

Bold and italics indicate statistical significance

The dependent variables in the separate regressions for the percentage of monitoring time with ICP > 20 mmHg and CPP < 60 mmHg were log_10_-transformed due to skewness of data. The hematoma volume was denoted as 0 mL in the analyses if the patient did not have that type of intracranial hemorrhagic lesion type. Regression 1; ICP > 20 mmHg, *R*^2^ = 0.18, ANOVA *p* < 0.001. Regression 2; CPP < 60, *R*^2^ = 0.19 ANOVA *p* < 0.001. Regression 3; PRx, *R*^2^ = 0.12, ANOVA *p* < 0.001

*ASDH* acute subdural hematoma, *CPP* cerebral perfusion pressure, *EDH* epidural hematoma, *ICP* intracranial pressure, *IVH* intraventricular hemorrhage, *PRx* pressure reactivity index, *tSAH* traumatic subarachnoid hemorrhage

^a^*p* < 0.05; ^b^*p* < 0.01; ^c^*p* < 0.001

^*^The extent of basal cistern compression was assessed as an ordinal variable (1/open, 2/compressed, 3/obliterated)

### Intracranial hemorrhage type, size, mass effect, and evolution in relation to clinical outcome

In univariate analyses, greater EDH size (*r* = 0.20, *p* < 0.001) was associated with higher GOS-E, whereas larger ASDH (*r* =  − 0.19, *p* < 0.001), presence of tSAH (*r* =  − 0.13, *p* < 0.05), larger IVH (*r* =  − 0.13, *p* < 0.05), larger contusions (*r* =  − 0.17, *p* < 0.001), greater midline shift (*r* =  − 0.18, *p* < 0.001), and more compressed basal cisterns (*r* =  − 0.23, *p* < 0.001) were associated with lower GOS-E (Table 3). In a multiple logistic regression for favorable outcome, a larger ASDH size (OR (95% CI) = 0.99 (0.98–0.99), *p* < 0.05), presence of tSAH (OR (95% CI) = 0.38 (0.20–0.74), *p* < 0.01), greater contusion size (OR (95% CI) = 0.99 (0.98–1.00), *p* < 0.05) and obliterated basal cisterns (OR (95% CI) = 0.05 (0.01–0.41), *p* < 0.01) in addition to older age (OR (95% CI) = 0.97 (0.95–0.98), *p* < 0.01), and lower GCS M at admission (OR (95% CI) 1.55 (1.22–1.96), *p* < 0.001) were associated with a lower rate of favorable outcome (Table 6).

**Table 6:** Intracranial hemorrhage type, size, mass effect, and evolution in relation to favorable outcome—a multiple logistic regression analysis

Variables	OR (95% confidence interval)	*p*-value
Age (years)	***0.97 (0.95–0.98)***	** < *0.001***
GCS M (scale)	***1.55 (1.22–1.96)***	** < *0.001***
Pupillary abnormality (yes)	0.56 (0.29***–***1.07)	0.08
EDH (mL)	1.01 (0.99***–***1.02)	0.53
ASDH (mL)	***0.99 (0.98–0.99)***	***0.04***
tSAH (yes)	***0.38 (0.20–0.74)***	***0.004***
IVH (mL)	0.95 (0.89***–***1.00)	0.06
Contusion (mL)	***0.99 (0.98–1.00)***	***0.04***
Midline shift (mm)	0.99 (0.91***–***1.06)	0.70
Basal cisterns (open—reference)		
Compressed (yes)	0.63 (0.26***–***1.54)	0.31
Obliterated (yes)	***0.05 (0.01–0.41)***	***0.006***
Hemorrhage evolution (stable—reference)		
Progression (yes)	1.71 (0.88***–***3.32)	0.12
Evacuation (yes)	2.15 (0.87***–***5.31)	0.10

Bold and italics indicate statistical significance. Nagelkerke = 0.33

The hematoma volume was denoted as 0 mL in case the analyses if the patient did not have that type of intracranial hemorrhagic lesion type

*ASDH* acute subdural hematoma, *EDH* epidural hematoma, *GCS M* Glasgow Coma Scale Motor score, *IVH* intraventricular hemorrhage, *OR* odds ratio, *tSAH* traumatic subarachnoid hemorrhage

## Discussion

In this study, including 385 patients with moderate to severe TBI, we found that tSAH and hemorrhagic progression predicted more ICP problems during the first week post-injury. Hemorrhagic progression and the need for emergency neurosurgery were also associated with more CPP insults, whereas severe mass effect with compressed/obliterated basal cisterns independently predicted a more disturbed cerebral pressure autoregulation. Obliterated basal cisterns, presence of tSAH, and larger ASDHs and contusions were independently associated with worse clinical outcome. Although these results were to some extent expected, our study provides a granular analysis on the effect of specific intracranial hemorrhage lesion type, mass effect, and evolution in the early course on clinical trajectories and neurological recovery. Future efforts should aim to integrate such data into more sophisticated machine learning models to aid the clinician to better anticipate emerging secondary insults and to more accurately predict clinical outcome in TBI.

The patients with large EDHs were operated with craniotomy to a larger extent, reflecting the natural course of EDHs as rapidly evolving lesions that require emergency evacuation to avoid brain herniation [5]. However, they did not require last-tier ICP treatments (thiopental and DC) as often and were not more prone to develop ICP insults. Since the ICP monitor was generally inserted at the time of hematoma evacuation, these results primarily support that postoperative ICP problems were not very frequent, possibly thanks to timely surgery before secondary brain injury had occurred. Presence of/larger EDHs was associated with favorable outcome in univariate analyses but not in the multiple regression analysis. Since an EDH is extra-cerebral and not primarily a cerebral injury, it is a more treatable lesion than other traumatic intracranial hemorrhagic types, in case of timely surgery. Consistent with our results, EDH has generally been associated with favorable outcome (as opposed to other injury types) in cohorts of moderate to severe TBI and is considered beneficial, e.g., in the Rotterdam, Helsinki, and Stockholm radiological scoring systems [17, 29].

Not surprisingly, those with larger ASDH volumes more often required craniotomy, but, in similarity with larger EDHs, they were not more prone to require last-tier ICP treatments or develop more ICP insults. Patients with larger ASDHs were generally older (*r* = 0.41, *p* < 0.001) and therefore more likely tolerated larger hematomas due to certain brain atrophy, which could also explain why larger ASDH size did not independently translate into more ICP problems. Also, if brain swelling occurred during evacuation of the ASDH, the bone flap was usually not repositioned, an action that likely reduced postoperative ICP problems in many severe ASDH cases. Interestingly, larger ASDHs were independently associated with less episodes of low CPP, even after adjustment for age as a potential confounder for high ABP and low ICP. Furthermore, we only found a univariate association between larger ASDH size and higher PRx, but not in multiple linear regressions. The association was likely attenuated after adjustment by higher age which correlated with both high PRx and large ASDHs. ASDHs have been associated with higher PRx in some [34], but not all studies [35]. It has been suggested that acceleration/deceleration injury mechanism could be a driver of cerebral pressure autoregulatory disturbances (high PRx) [34, 35] and it is possible that differences in the underlying mechanism for the ASDH (elderly with falls vs. young patients in road traffic accidents) are crucial for the association with PRx. Large ASDHs are on the one hand extra-cerebral and potentially treatable if evacuated in time, but they are also associated with initiating underlying hemispheric injury including ischemia, reperfusion injury, and edema predisposing for secondary brain injury [21]. The combination of these factors likely explained its independent association with unfavorable outcome, consistent with previous studies [17, 29].

tSAH was common in the study population and occurred in 73% of the cases. tSAH typically includes a diffuse spread of small amounts of blood in the subarachnoid space not causing any mass effect, per se, which likely explained the lower rate of craniotomies in this group. However, still, tSAH correlated with the need to proceed with DC surgery and was also independently associated with more ICP problems. For example, tSAH has previously shown to be an indicator of evolving brain injury such as contusions that have not yet blossomed on early CT scans [2, 12]. Furthermore, there was no association between tSAH and PRx. This was to some extent surprising, since tSAH has been associated post-traumatic vasospasm [1] which likely overlaps with the pressure autoregulatory capacity to some extent. However, it is possible that our tSAH assessments (present or absent) were too crude to fully assess the burden of this lesion type. Currently, the literature is also conflicting, with studies both in favor [34] and against [35] an association between tSAH and high PRx. tSAH was also independently associated with unfavorable outcome. Although isolated tSAH in mild TBI cohort is a favorable finding, previous studies support that the opposite is true in moderate to severe TBI [14] and, e.g., tSAH carries a negative prognostic finding of worse outcome in the Rotterdam score [17]. In addition, IVHs were associated with a lower incidence of craniotomy and were independently associated with a lower percentage of ICP problems, possibly as it is rather a condition that is more effectively treated with an EVD in case cerebrospinal fluid circulation disturbances ensue.

Larger contusion volumes were associated with thiopental and DC treatment, but not with craniotomy. This was probably partly explained by the fact that many contusions were quite small (median 7 mL) without midline shift and also that total volume was based on the sum of potentially multiple, scattered contusions more resembling a diffuse injury. In addition, for the cases with more diffuse spread of contusions involving extensive areas of the brain, DC might have been preferred over evacuation [16]. Also, e.g., scattered multiple contusions with ICP problems without midline shift, thiopental might have been a feasible option for ICP control. Contusion volumes were not associated with cerebral pressure autoregulatory disturbances, consistent with some previous studies [34]. However, in more granular studies, Zeiler et al. established a potential link between autoregulatory disturbances and the risk of progression of peri-contusional edema [19, 20]. However, in this study, we assessed the total contusion volume and rather assessed PRx during the entire monitoring than between the radiological scans which could explain the discrepant findings. Lastly, contusions are per definition an intracerebral injury and greater size implies more extensive neurological injury. Consistently, greater contusion size was independently associated with worse outcome in this study.

Not surprisingly, indicators of significant mass effect, i.e., midline shift and compressions of basal cisterns, were associated with a worse clinical trajectory. Both correlated with a higher frequency of craniotomy and DC surgery. These measures of mass effect were not independently associated with more ICP insults, most likely since such malignant early signs of brain herniation required aggressive emergency neurosurgery (craniotomy and DC) which alleviated ICP post-operatively when it was monitored and analyzed. Still, compressed/obliterated basal cisterns were independently associated with disturbances in cerebral pressure autoregulation during the first 7 days post-injury, which was also found in a previous study [35]. This has been suggested to reflect severe diffuse brain injury caused by acceleration/deceleration forces [35] that may in turn increase the susceptibility for secondary injury due to cerebral ischemia and disturbances in energy metabolism [3, 24]. Obliterated basal cisterns were also independently associated with poor outcome, consistent with the Marshall classification and the Rotterdam and Helsinki scoring system [17, 18, 29]. The inclusion of this radiological measure of brain herniation in the multiple regression likely explained why the association between unreactive pupils and worse outcome was attenuated.

Furthermore, intracranial hemorrhage progression was independently associated with more ICP and CPP insults. This probably both reflected that these patients experienced greater intracranial hemorrhagic volumes leading to higher ICP, but also implied an evolving process between the CTs while ICP monitoring might have already been started and therefore captured in this data. However, hemorrhage progression and emergency neurosurgery were not independent predictors of worse outcome, as this was rather explained by specific hemorrhagic lesion types and mass effect.

On balance, classifications of intracranial hemorrhagic lesion types, sizes, mass effect, and evolution from early CT scans post-injury may be of value for predicting the clinical trajectories and development of secondary insults of important NIC variables such as ICP, CPP, and PRx. In future efforts, it may be of value to integrate such data into machine learning models for more granular predictions of these secondary insults during NIC [7] and long-term clinical outcome [8]. We also found that secondary ICP and CPP insults were rare, occurred in median in less than 5% of the monitoring time, and was overall only weakly associated with the majority of the intracranial hemorrhage types and their volumes. Thus, attentive NIC was important to control these physiological parameters under all circumstances, despite the great heterogeneity on early imaging in lesion types, volumes, and mass effect.

### Methodological considerations

The strengths of this study was the relatively large patient cohort with a granular methodology including both very accurate radiological volume estimations of traumatic intracranial hemorrhages and high-frequency data for the main NIC variables of cerebral physiology. Although intracranial lesion features in relation to clinical management has been investigated previously, by, e.g., Wintermark et al. [9, 33], those studies were oriented from an emergency department point of view (in relation to discharge, admission, need for neurosurgery, and high risk for mortality) whereas this study was focused on those admitted to the NIC and the role of neurosurgical monitoring and treatment.

The main limitations were the retrospective and single-center design of this study, which limits the external validity. Furthermore, although we strived for a high adherence to the local treatment protocol within our center, there was most likely some variation in clinical decision-making among the colleagues. This probably had some effect on the association between intracranial hemorrhage features and the treatment variables, NIC course, and outcome. Due to technical difficulties, we abstained from further analyses of tSAH volume. The analysis of IVH may to some extent be unreliable since the hemorrhage could have been diluted to various extent in the cerebrospinal fluid and we did not control for any potential difference in Hounsfield units of the IVH volume between the scans. Third, in further studies, more elaborate analyses on specific injury patterns of isolated hemorrhagic lesions and various mixed injury combinations may yield a deeper understanding on the clinical course during NIC and outcome. Similarly, it would be interesting to proceed with more granular studies of hemorrhagic volumes in relation to cerebral physiology in relation to specific interventions such as hematoma evacuation, based on pre- and postoperative CT scans. Lastly, significant hemorrhage evolution has previously been defined in many different ways (e.g., absolute vs. relative increase in hemorrhage volume). We defined it as an absolute hemorrhagic increase with 6 mL, similar to Shin et al. and a previous study by our group [23, 25]. This threshold was set relatively high to really capture large and truly clinically significant hemorrhage progression.

## Conclusions

TBI is a heterogeneous disease with several different types of intracranial hemorrhagic lesions of various sizes, mass effect, and evolution. It is generally challenging to predict the clinical course and outcome. This study provides granular radiological analyses of lesion specific variables (such as lesion type, size, and progression) in relation to high-frequency variables of cerebral physiology (e.g., ICP burden and PRx status) and long-term clinical outcome. Future efforts should to integrate such granular data into more sophisticated machine learning models to aid the clinician to better anticipate emerging secondary insults and to predict clinical outcome.

## Data Availability

Data are available upon reasonable request.
